# CpG-DNA exerts antibacterial effects by protecting immune cells and producing bacteria-reactive antibodies

**DOI:** 10.1038/s41598-018-34722-y

**Published:** 2018-11-02

**Authors:** Te Ha Kim, Dongbum Kim, Avishekh Gautam, Heesu Lee, Min Hyung Kwak, Min Chul Park, Sangkyu Park, Guang Wu, Bok Luel Lee, Younghee Lee, Hyung-Joo Kwon

**Affiliations:** 1 Department of Microbiology, College of Medicine, Hallym University, Chuncheon, 24252 Republic of Korea; 2 Center for Medical Science Research, College of Medicine, Hallym University, Chuncheon, 24252 Republic of Korea; 3 Department of Biochemistry, College of Natural Sciences, Chungbuk National University, Cheongju, 28644 Republic of Korea; 4 Global Research Laboratory of Insect Symbiosis, College of Pharmacy, Pusan National University, Pusan, 46241 Republic of Korea

## Abstract

CpG-DNA activates various immune cells, contributing to the host defense against bacteria. Here, we examined the biological function of CpG-DNA in the production of bacteria-reactive antibodies. The administration of CpG-DNA increased survival in mice following infection with methicillin-resistant *S. aureus* and protected immune cell populations in the peritoneal cavity, bone marrow, and spleen. CpG-DNA injection likewise increased bacteria-reactive antibodies in the mouse peritoneal fluid and serum, which was dependent on TLR9. B cells isolated from the peritoneal cavity produced bacteria-reactive antibodies *in vitro* following CpG-DNA administration that enhanced the phagocytic activity of the peritoneal cells. The bacteria-reactive monoclonal antibody enhanced phagocytosis *in vitro* and protected mice after *S. aureus* infection. Therefore, we suggest that CpG-DNA enhances the antibacterial activity of the immune system by protecting immune cells and triggering the production of bacteria-reactive antibodies. Consequently, we believe that monoclonal antibodies could aid in the treatment of antibiotic-resistant bacterial infections.

## Introduction

The innate immune system is the first line of host defense against invading pathogens and potentially harmful agents^1^. Toll-like receptor 9 (TLR9), an important pathogen recognition receptor, detects and binds bacterial DNA, leading to immunomodulatory effects in the host^2^. Bacterial DNA and synthetic oligonucleotides containing CpG dinucleotide motifs (CpG-DNA) activate various cells, stimulating cell proliferation and the production of Th1-mediated cytokines through the stimulation of TLR9^3–6^. In addition, CpG-DNA triggers the proliferation and differentiation of B cells, and the production of T cell-independent polyclonal antibodies^7^. Using TLR9 knockout mice, several investigators discovered that TLR9 exhibits a protective role against select bacterial infections, including *Mycobacterium tuberculosis*, *Mycobacterium avium*, *Klebsiella pneumoniae*, *Legionella pneumophila*, *Acinetobacter baumannii*, and methicillin-resistant *Staphylococcus aureus* (MRSA)^8–13^. Several studies also reported that the administration of CpG-DNA in both *in vitro* and *in vivo* model systems provided protection against bacterial infection, such as *Listeria monocytogenes*, *Francisella tularensis*, *Klebsiella pneumoniae*, *Salmonella typhimurium*, and *Staphylococcus aureus* (*S. aureus*)^14–18^.

CpG-DNA-activated Thy1.2+ dendritic cells exhibit protective roles against *Listeria monocytogenes* infection in murine models via the secretion of IFN-γ^14^. Similarly, the protective role of CpG-DNA against *Klebsiella pneumoniae* infection also requires the production of IFN-γ^16^. In osteoblast-like cell lines, the antibacterial effects of CpG-DNA against *S. aureus* infection involve TLR9 and the induction of oxidative mediators^18^. Further, CpG-DNA treatment increases the induction of phagocytosis in *S. aureus*-infected RAW264.7 macrophage cells and mouse peritoneal macrophages through the JNK/P38 signaling pathway^19^. However, the biological effects of the antibodies produced following CpG-DNA stimulation are not known.

*S. aureus* is a major pathogen in the etiology of many infectious diseases ranging from mild skin and soft tissue inflammation to life-threatening diseases such as sepsis, endocarditis, and pneumonia^20,21^. Alarmingly, the treatment of these infectious diseases with multiple different antibiotics has been complicated by the emergence of MRSA strains^22^. Because of the reduced efficacy of antibiotics and increased emergence of MRSA strains, novel strategies for the treatment of MRSA infections are urgently needed. To this end, the development of vaccines and protective antibodies could provide valuable alternative strategies to combat MRSA infections^23–25^. Recently, researchers developed a monoclonal antibody that is reactive to *S. aureus* surface proteins and demonstrated its protective activity in murine models^26^.

Here, we show that the administration of CpG-DNA in the mouse peritoneal cavity enhances resistance against *S. aureus* infection, and that the antibodies induced by CpG-DNA in the mouse peritoneal cavity exhibit protective functions against *S. aureus* infection via an antibody-dependent phagocytosis pathway. This novel CpG-DNA function provides insight into the antibacterial effects of CpG-DNA and suggests that the monoclonal antibody produced could be useful for the development of a novel strategy for treating MRSA infections.

## Results

### Administration of CpG-DNA enhances survival in mice and facilitates bacterial clearance in tissues after *S. aureus* MW2 infection

**Figure 1:**
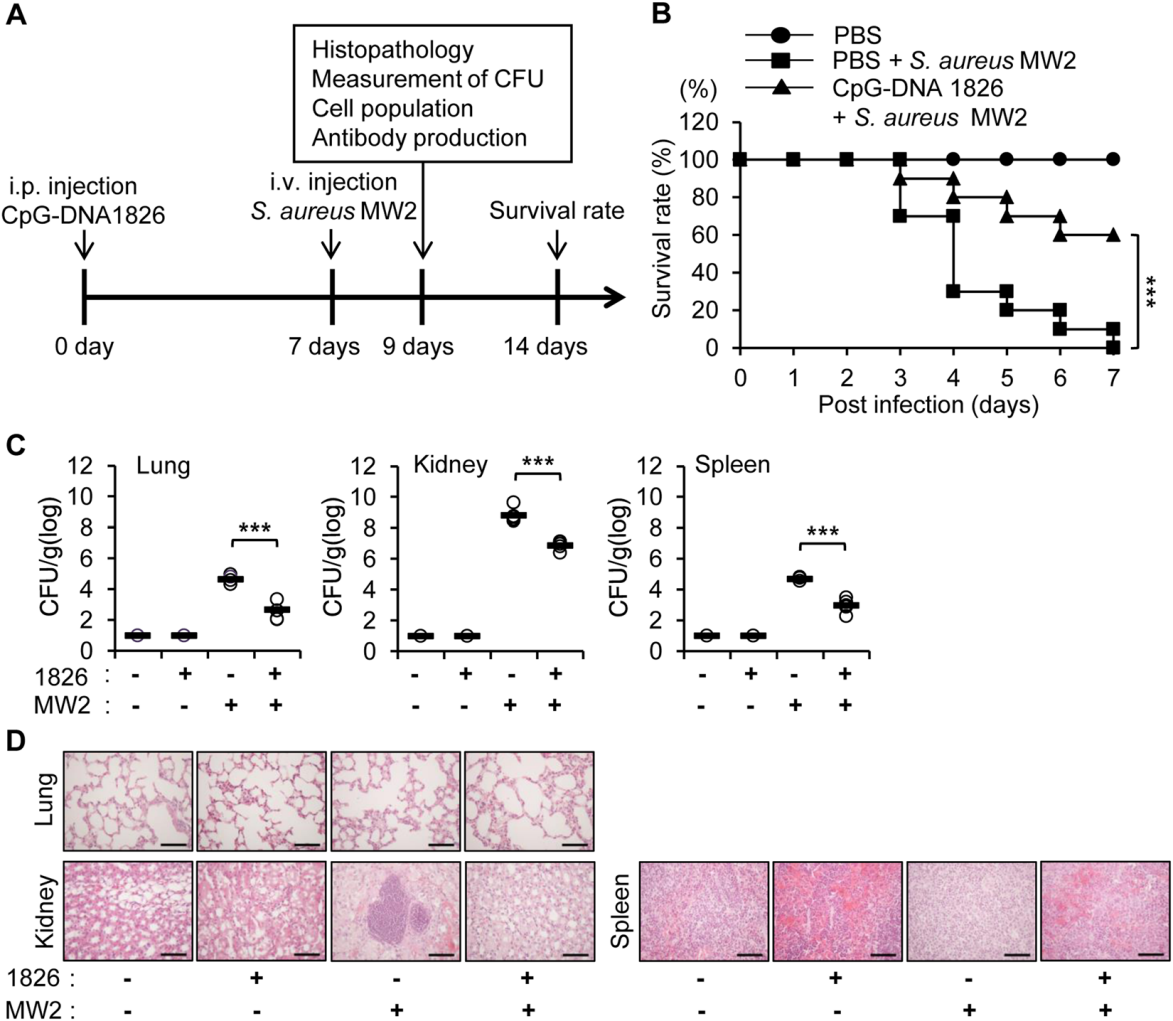
CpG-DNA protects mice from *S. aureus* MW2 infection. (**A**) Schematic diagram of the experimental process. BALB/c mice were administered CpG-DNA 1826 via an i.p. injection (2.5 mg/kg mouse). After 7 days, the mice were i.v. injected with *S. aureus* MW2 (1 × 10^7^ CFU). **(B)** Survival of the mice was recorded for 7 days after *S. aureus* MW2 infection. The percentage of surviving mice in each treatment group is shown (n = 10/group). **(C)** Two days after *S. aureus* MW2 infection, the mice were sacrificed, and the indicated tissues were removed and homogenized in PBS. The homogenates (n = 5/group) were diluted and plated on agar plates to measure *S. aureus* MW2 colony forming units (CFU). **(D)** Histopathology of the indicated tissues two days after infection. Scale bar, 10 μm. 1826, CpG-DNA 1826; MW2, *S. aureus* MW2. The results presented are representative of three independent experiments. ***p < 0.0005.

*S. aureus* MW2 is a community-associated MRSA strain possessing virulence factors that, when secreted, caused several fatal infections^27,28^. To determine whether CpG-DNA can protect against *S. aureus* MW2 infection, we performed experiments using BALB/c mice according to the procedure depicted in Fig. 1A. The BALB/c mice received an intraperitoneal (i.p.) injection of PBS or CpG-DNA 1826 (2.5 mg/kg mouse). After 7 days, the mice received an intravenous (i.v.) injection of *S. aureus* MW2 (1 × 10^7^ colony forming units (CFU)), and survival rates were monitored for 7 days. Compared to the mice that only received *S. aureus* MW2, the survival rate of the mice pre-treated with CpG-DNA prior to *S. aureus* MW2 infection was 50% greater (10% vs 60%, Fig. 1B).

Two days after *S. aureus* MW2 infection, the lungs, kidney, and spleen were excised to assess bacterial burden. All of the tissues tested were infected by bacteria, with the highest CFU detected in the kidney. However, the tissue bacterial loads were all decreased by CpG-DNA 1826 pre-treatment (Fig. 1C). Next, the histopathology of each tissue was examined. Abscesses were observed in the kidneys after bacterial infection; however, no abscesses were detected when the mice were pre-treated with CpG-DNA 1826 before infection (Fig. 1D). Therefore, these results suggest that the prophylactic injection of CpG-DNA into the peritoneal cavity increased survival and enhanced bacterial clearance in mice subsequently infected with *S. aureus* MW2.

### CpG-DNA protects and modulates cell populations in the peritoneal cavity, spleen, and bone marrow

**Figure 2:**
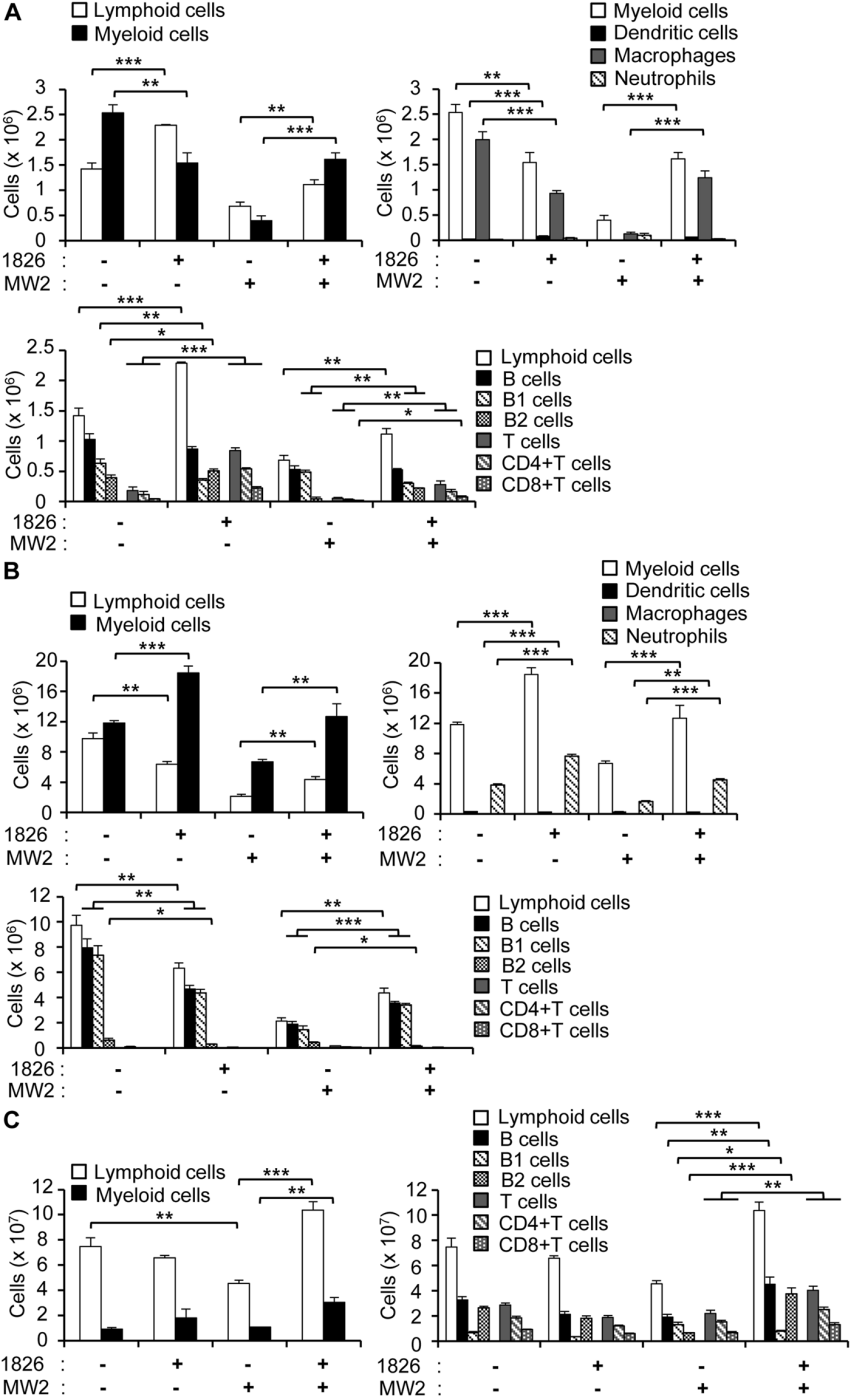
Changes in the cell populations in the mouse peritoneal cavity, spleen, and bone marrow after *S. aureus* MW2 infection. (**A**–**C)** BALB/c mice were i.p. injected with PBS or CpG-DNA 1826. After 7 days, the mice were i.v. injected with PBS or *S. aureus* MW2 (1 × 10^7^ CFU). Two days after *S. aureus* MW2 infection, the mice were sacrificed. The peritoneal cavity cells, splenocytes, and bone marrow cells were harvested and stained with fluorescence-conjugated antibodies to analyze the cell populations by flow cytometry. **(A)** Peritoneal cavity cells. **(B)** Bone marrow cells. **(C)** Splenocytes. n = 3/group. 1826, CpG-DNA 1826. MW2, *S. aureus* MW2. The results presented are representative of three independent experiments. *p < 0.05, **p < 0.005, ***p < 0.0005.

To investigate the mechanisms underlying the apparent protective effects of CpG-DNA pre-treatment against *S. aureus* MW2 infection, we analyzed cell populations in the peritoneal cavity, spleen, and bone marrow and found that lymphoid (B and T cells) and myeloid cells (macrophages, dendritic cells, and neutrophils) were altered by CpG-DNA pre-treatment and *S. aureus* MW2 infection in the different tissues (Fig. 2).

In the peritoneal cavity, the ratio of myeloid and lymphoid cells was reversed by CpG-DNA treatment (Fig. 2A). The myeloid cell population in the peritoneal cavity was decreased by CpG-DNA administration (61% compared to the PBS control), whereas the population of lymphoid cells was increased (160% compared to the PBS control). The myeloid cell population was primarily comprised of F4/80^+^CD11b^+^ macrophages. Although the population of F4/80^−^CD11c^+^ dendritic cells was very small, CpG-DNA administration caused a 3-fold increase in the cell number compared to the PBS control. In the case of the lymphoid cells, the increase in the T cell population, including CD4^+^ and CD8^+^ T cells, was marked (4.6-fold compared to the PBS control). As well, similar increases were observed in the total number of B cells. A detailed analysis of the B cell population revealed that B1 (CD23^−^) cells were decreased and B2 (CD23^+^) cells were slightly increased following CpG-DNA treatment. Following infection with *S. aureus* MW2, all of the cells markedly decreased except neutrophils, but pre-treatment with CpG-DNA resulted in cell populations that were similar to the untreated control, despite lower total cell numbers (Fig. 2A).

In the bone marrow, the population of myeloid cells increased (150% compared to the PBS control), whereas the lymphoid cell population decreased (65% compared to the PBS control) in response to CpG-DNA (Fig. 2B). Further, the population of myeloid cells was primarily comprised of F4/80^−^Gr-1^+^ neutrophils. The majority of the lymphoid cells were B cells, especially B1 cells. In contrast to the peritoneal cavity, the ratio of B1 and B2 cells was not changed by CpG-DNA treatment. *S. aureus* MW2 infection induced a marked decrease in all of the cell populations, but the decreases were prevented by pre-treatment with CpG-DNA (Fig. 2B).

In the spleen, CpG-DNA administration did not induce any significant changes in the cell populations (Fig. 2C). However, a decrease in lymphoid cells was observed (60% compared to the PBS control) following infection with *S. aureus* MW2. When the mice were pre-treated with CpG-DNA before bacterial infection, the number of lymphoid cells was greater than the PBS control. Compared to *S. aureus* MW2 infection alone, pre-treatment with CpG-DNA resulted in greater than 2-fold increases in both lymphoid and myeloid cells (Fig. 2C).

Based on these results, we concluded that *S. aureus* MW2 infection induces decreases in the total numbers of myeloid and lymphoid cells in the peritoneal cavity, bone marrow, and spleen. However, pretreating the mice with CpG-DNA generally prevented the apparent decreases in immune cells following infection. This phenomenon suggests that the observed cell numbers following CpG-DNA treatment may be related to the mouse survival rates. Most importantly, CpG-DNA treatment shifted the population of immune cells in the peritoneal cavity toward increased macrophages and adaptive immune cells such as B2 and T cells (Fig. 2A). Therefore, we propose that the cells in the peritoneal cavity may be the primary regulator of the antibacterial effects of CpG-DNA in our experimental system.

### Antibodies binding to bacteria are generated by CpG-DNA injection *in vivo* and *in vitro*

**Figure 3:**
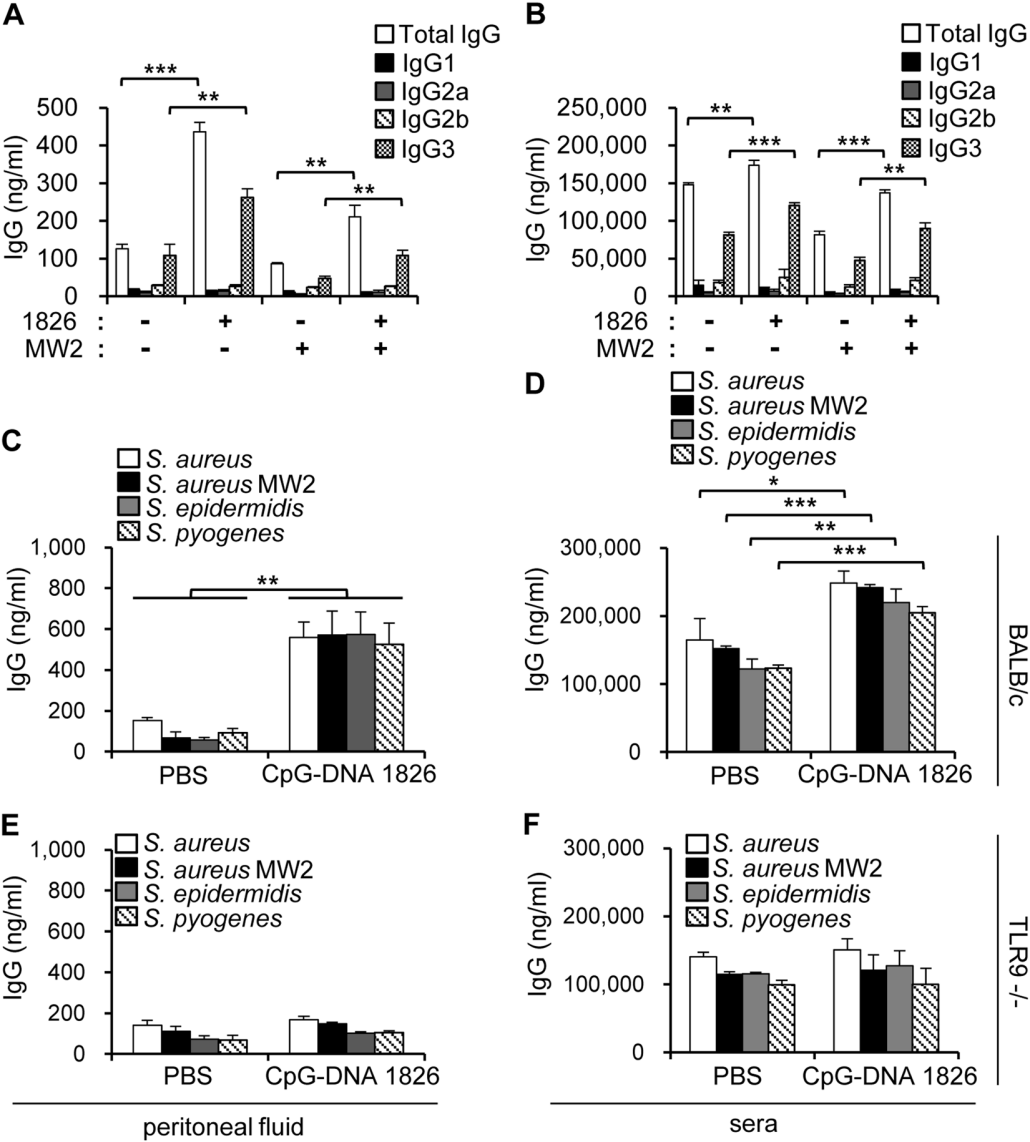
Production of bacteria-reactive antibodies in the mouse peritoneal cavity and serum following the administration of CpG-DNA 1826. (**A**,**B**) BALB/c mice were i.p. injected with CpG-DNA 1826. After 7 days, the mice were i.v. injected with *S. aureus* MW2 (1 × 10^7^ CFU). Two days after bacterial infection, the peritoneal fluid and sera were collected from the mice. Bacteria-reactive antibodies in the peritoneal fluid (**A**) and sera (**B**) were captured using *S. aureus* MW2 coated plates (n = 3/group) and the concentrations of total IgG and each IgG isotype were measured by ELISA. 1826, CpG-DNA 1826. MW2, *S. aureus* MW2. (**C**–**F)** BALB/c (**C**,**D**) and TLR9^−/−^ (**E**,**F**) mice were i.p. injected with CpG-DNA 1826. Seven days after the administration of CpG-DNA 1826, supernatants were collected from the peritoneal fluid (**C**,**E**) and sera (**D**,**F**). To measure the amounts of antibodies reactive to Gram-positive bacteria, the indicated bacteria were added to poly-L-lysine-coated plates and the amount of bound IgG was determined by ELISA (n = 3/group). The results presented are representative of three independent experiments. *p < 0.05, **p < 0.005, ***p < 0.0005.

Because the administration of CpG-DNA triggered B cell proliferation and differentiation and the production of T cell-independent polyclonal antibodies^7^, we examined the IgG titer in the peritoneal fluid and serum at various time points after i.p. injection of CpG-DNA 1826 to confirm antibody production. Based on our observation that the injection of CpG-DNA enhanced survival against *S. aureus* MW2 infection, we believed it was likely that some bacteria-reactive antibodies could be induced by the injection of CpG-DNA 1826 in the peritoneal cavity. We harvested peritoneal fluid and serum from mice following the i.p. injection of CpG-DNA 1826 and i.v. infection with *S. aureus* MW2 (Fig. 1A) and measured total levels of IgG and IgG isotypes reactive to *S. aureus* MW2 using plates coated with *S. aureus* MW2. CpG-DNA alone induced a drastic increase in reactive total IgG in the peritoneal fluid, but there was comparatively small change in the sera. Infection with *S. aureus* MW2 caused a decrease in the production of reactive IgG both in the peritoneal fluid and serum. However, pre-administration of CpG-DNA 1826 prior to bacterial infection induced a significantly increased production of reactive IgG. The IgG3 isotype was the most abundant and the amount of *S. aureus* MW2-reactive IgG3 isotype was significantly increased in the CpG-DNA-treated group (Fig. 3A,B).

To further investigate the antibodies induced by CpG-DNA administration, the mice received an i.p. injection of PBS or CpG-DNA 1826 and the peritoneal fluid and serum were analyzed after 7 days. To determine if the CpG-DNA-induced IgG could bind various Gram-positive bacterial species, we performed an ELISA assay using plates coated with *S. aureus*, *S. aureus* MW2, *S. epidermidis*, and *S. pyogenes*. The ELISA results indicated that the levels of each bacteria-reactive IgG were increased in the peritoneal cavity and sera following the injection of CpG-DNA 1826 (Fig. 3C,D). To determine if treatment with CpG-DNA activated the TLR9 signaling pathway to produce bacteria-reactive antibodies, the same experiments were performed using BALB/c TLR9^−/−^ mice. No significant changes in the levels of bacteria-reactive antibodies were detected in the peritoneal fluid or sera of TLR9^−/−^ mice following the injection of CpG-DNA 1826 (Fig. 3E,F). Therefore, these data suggest that CpG-DNA induced the production of bacteria-reactive antibodies through the TLR9 signaling pathway.

**Figure 4:**
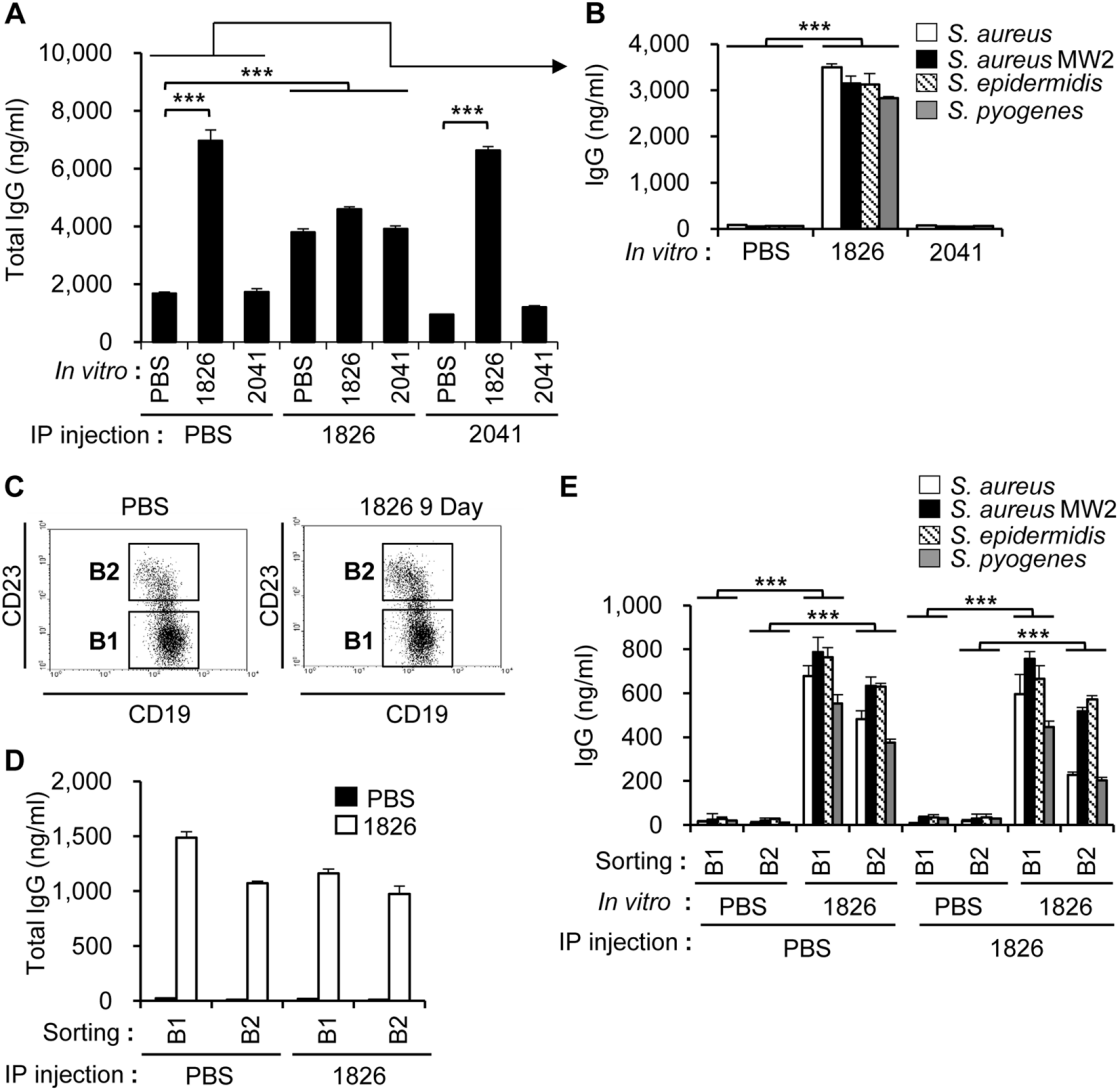
Production of bacteria-reactive antibodies in mouse peritoneal cavity cells following treatment with CpG-DNA 1826 *in vitro*. BALB/c mice were i.p. injected with PBS, CpG-DNA 1826, or non-CpG-DNA 2041. **(A)** After 7 days, the cells from the peritoneal cavity were harvested and then stimulated with PBS, CpG-DNA 1826, or non-CpG-DNA 2041. After 48 h, the cell culture supernatants were collected, and the amounts of total IgG were determined by ELISA (n = 3/group). **(B)** The peritoneal cells from PBS-injected mice were stimulated with PBS, CpG-DNA 1826, or non-CpG-DNA 2041. To measure the amounts of antibodies reactive to Gram-positive bacteria, the indicated bacteria were added to poly-L-lysine-coated plates and the cell culture supernatants were applied. The amount of bound IgG was determined by ELISA (n = 3/group). **(C)** After the i.p. administration of PBS or CpG-DNA 1826 to BALB/c mice, B1 and B2 cells were isolated from the peritoneal cavity using a FACSAria^TM^ II system and fluorescence-labeled anti-mouse CD19 and anti-mouse CD23 antibodies. **(D,E)** Isolated B1 and B2 cells from the peritoneal cavity were stimulated with PBS or CpG-DNA 1826. After 48 h, the cell culture supernatants were collected and the amount of total IgG **(D)** and the antibodies reactive to Gram-positive bacteria **(E)** were determined by ELISA (n = 3/group). 1826, CpG-DNA 1826. 2041, non-CpG-DNA 2041. The results presented are representative of three experiments. ***p < 0.0005.

To determine whether bacteria-reactive antibodies could be induced *in vitro* by CpG-DNA, immune cells were harvested from the peritoneal cavity of the mice, stimulated *in vitro* with CpG-DNA 1826 and non-CpG-DNA 2041, and the antibody levels were measured in the cell culture supernatants. As shown in Fig. 4A, general IgG production was significantly increased in response to CpG-DNA 1826 compared to PBS control or non-CpG-DNA 2041. When the mice were first primed with CpG-DNA *in vivo* and the peritoneal cavity cells were stimulated *in vitro*, basal IgG production was enhanced, but the added effect of CpG-DNA treatment was very weak (Fig. 4A). To determine the binding capability of the antibodies secreted from the *in vitro*-cultured peritoneal cells, we measured bacteria-reactive IgG levels using plates coated with the four Gram-positive bacteria species. Bacteria-reactive IgG was significantly increased in response to CpG-DNA, but the reactive antibody levels detected in response to PBS or non-CpG-DNA were very low (Fig. 4B).

Generally, antibody production is associated with the presence of mature B cells, such as plasma B cells. To investigate which B cells were secreting bacteria-reactive antibodies in response to CpG-DNA, an i.p. injection of CpG-DNA 1826 was administered to the mice. Nine days after the injection of CpG-DNA 1826, we harvested immune cells from the peritoneal cavities of the mice, sorted CD23^−^CD19^+^ B cells (B1 cells) and CD23^+^CD19^+^ cells (B2 cells) from peritoneal lymphocytes (Fig. 4C) and stimulated the cells with CpG-DNA 1826 *in vitro*. Both the B1 and B2 cells secreted increased amounts of IgG in response to CpG-DNA stimulation (Fig. 4D). However, the IgG levels produced by B cells *in vitro* were decreased when the mice were pre-treated with CpG-DNA prior to the isolation of the peritoneal cavity cells, suggesting that the CpG-DNA modulated B cell activity (Fig. 4D). Assays of each bacteria-reactive IgG, measured using Gram-positive bacteria-coated plates, suggested that the production of bacteria-reactive IgG was increased by CpG-DNA stimulation *in vitro* and that priming with CpG-DNA *in vivo* modulated antibody production (Fig. 4E). Taken together, these results indicate that both B1 and B2 cells produced bacteria-reactive antibodies in the peritoneal cavity in response to the administration of CpG-DNA.

### Bacteria-reactive antibodies in the peritoneal cavity induced by CpG-DNA enhance phagocytosis

**Figure 5:**
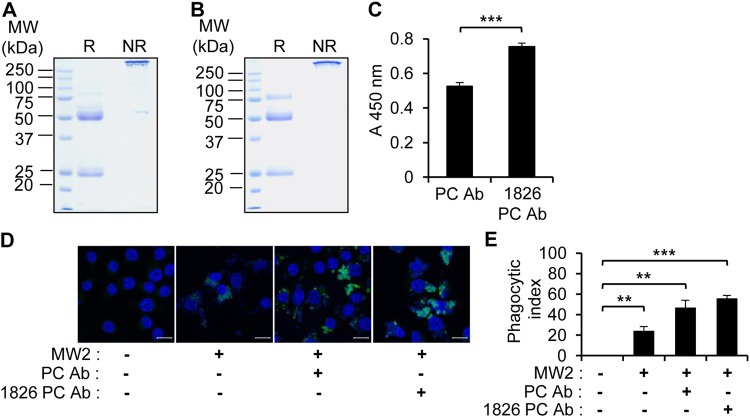
Enhancement of phagocytosis by CpG-DNA 1826-induced polyclonal antibodies. (**A,B**) BALB/c mice were i.p. injected with PBS (n = 80) **(A)** or CpG-DNA 1826 (n = 80) **(B)**. After 7 days, the peritoneal fluids were collected, and polyclonal antibodies were purified using Protein A affinity beads. The purified antibodies were prepared using reducing (R) or non-reducing (NR) sample buffer and subjected to SDS-PAGE and staining with Coomassie brilliant blue R-250 solution. **(C)** The ability of the antibodies (10 μg/ml) to bind *S. aureus* MW2 was measured by ELISA. PC Ab, purified antibodies from PBS-administered peritoneal cavity. 1826 PC Ab, purified antibodies from CpG-DNA 1826-administered peritoneal cavity. The absorbance was read at 450 nm. **(D)** FITC-labeled *S. aureus* MW2 (3 × 10^8^ CFU/ml) was incubated with PBS, PC Ab, or 1826 PC Ab (10 μg/ml) for 1 h then added to RAW 264.7 cells. After 1 h, the RAW 264.7 cells were washed with PBS, fixed, and stained with Hoechst No. 33258 to visualize the nuclei (blue). Confocal images revealed the phagocytosis of *S. aureus* MW2. Scale bars, 10 μm. **(E)** The phagocytic index, or the number of FITC-labeled *S. aureus* MW2 cells taken by the macrophage, was analyzed (n = 3/group). The results presented are representative of three experiments. **p < 0.005, ***p < 0.0005.

We examined whether bacteria-reactive antibodies induced by CpG-DNA in the peritoneal cavity could enhance phagocytosis and consequently inhibit bacterial infection. We purified the polyclonal antibodies from the peritoneal fluid of PBS-injected mice (Fig. 5A) and CpG-DNA 1826-injected mice (Fig. 5B). The total amounts of IgG in the peritoneal cavity were increased approximately 2.5-fold by CpG-DNA 1826 administration compared to the PBS controls (7.5 μg/mouse versus 3 μg/mouse). The ability of the antibodies to bind *S. aureus* MW2 was also measured by ELISA. The results revealed increased binding of the antibodies induced by the administration of CpG-DNA 1826 to *S. aureus* MW2 than the antibodies from the PBS-injected mice (Fig. 5C).

Next, we investigated the phagocytic capacity of these antibodies. Fluorescein isothiocyanate (FITC)-labeled *S. aureus* MW2 was incubated with the purified antibodies and a phagocytosis assay was performed by confocal microscopy using RAW 264.7 mouse macrophage cells (Fig. 5D,E). The phagocytic index was increased in the presence of the antibodies.

### Antibacterial effect of the bacteria-reactive monoclonal antibody

**Figure 6:**
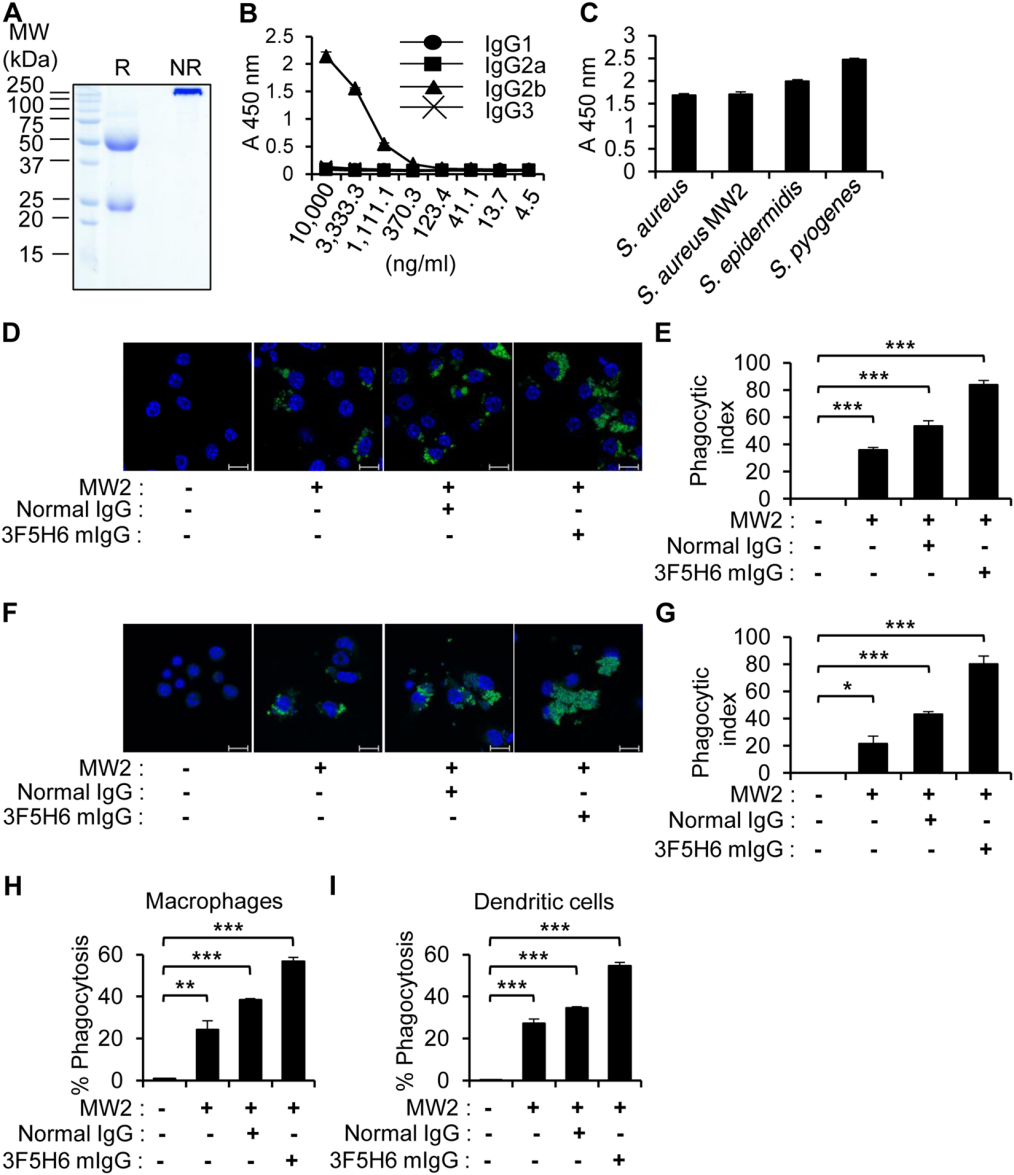
Enhanced phagocytosis induced by the monoclonal antibody produced by CpG-DNA 1826-stimulated mouse peritoneal cavity B cells. (**A**) Production of the bacteria-reactive monoclonal antibody. Ascites from mice induced by the 3F5H6 clone were isolated, and the resulting monoclonal antibody was purified by Protein A affinity column chromatography, subjected to SDS-PAGE, and stained with Coomassie brilliant blue R-250 solution. R, reducing. NR, non-reducing. (**B**) The isotype of the monoclonal antibody was determined by ELISA using *S. aureus* MW2-coated plates. (**C**) The bacteria-reactivity of the antibody was assessed by ELISA using plates coated with the indicated Gram-positive bacteria (n = 3/group). (**D**–**G**) FITC-labeled *S. aureus* MW2 cells (3 × 10^8^ CFU/mL) were incubated with PBS, normal mouse IgG, or 3F5H6 mIg (10 μg/mL) for 1 h then added to RAW 264.7 cells (**D,E**) and peritoneal cells (**F,G**) *in vitro*. After 1 h, the cells were washed with PBS, fixed, and stained with Hoechst No. 33258 to visualize the nuclei (blue). (**D,F**) Confocal images revealed phagocytosis of *S. aureus* MW2. Scale bars, 10 μm. MW2, *S. aureus* MW2. (**E,G**) The phagocytic index was analyzed (n = 3/group). (**H,I**) Phagocytosis was enhanced by the bacteria-reactive monoclonal antibody. FITC-labeled *S. aureus* MW2 cells (3 × 10^8^ CFU/mL) were incubated with normal mouse IgG or 3F5H6 mIgG (10 μg/mL) for 1 h and i.p. injected into BALB/c mice. After 1 h, peritoneal cells were harvested from the mice and stained with specific markers for macrophages (**H**) and dendritic cells (**I**). The phagocytic levels were analyzed by flow cytometry (n = 3/group). The results presented are representative of three experiments. *p < 0.05, **p < 0.005, ***p < 0.0005.

Based on our results, we believed that activated B cell clones induced by CpG-DNA might secrete antibodies harboring antibacterial activity, and that the antibodies might enhance phagocytosis via macrophages, dendritic cells, and neutrophils from the peritoneal cavity. To construct the B cell clone secreting bacteria-reactive antibodies induced by CpG-DNA, the mice were i.p. injected with CpG-DNA 1826. After 7 days, the peritoneal cells were harvested and fused with SP2/0 myeloma cells. We isolated the hybridoma clone, termed 3F5H6, which was secreting a monoclonal antibody reactive to *S. aureus* MW2. The monoclonal antibody (3F5H6 mIgG) purified from ascites was analyzed by SDS-PAGE (Fig. 6A). The 3F5H6 mIgG isotype was IgG2b (Fig. 6B), and the ability of 3F5H6 mIgG to bind several Gram-positive bacteria was confirmed by ELISA (Fig. 6C). Because IgG can non-specifically bind to *S. aureus* through Protein A, the same experiments were performed using a *S. aureus* Protein A deletion mutant (*S. aureus* Δ*spa)*. Normal IgG and 3F5H6 mIgG were reactive to *S. aureus* Δ*spa*, suggesting that the binding of normal IgG and 3F5H6 mIgG to *S. aureus* MW2 was specific (Supplementary Fig. S1).

To examine the effect of 3F5H6 mIgG on RAW 264.7 cell-mediated phagocytosis, FITC-labeled *S. aureus* MW2 was incubated with PBS, normal mouse IgG, or 3F5H6 mIgG (Fig. 6D,E). We also investigated the effect of 3F5H6 mIgG on mouse peritoneal cavity cell-mediated phagocytosis (Fig. 6F,G). The results indicated that the phagocytic activity of RAW264.7 cells and mouse peritoneal cavity cells was increased in the presence of 3F5H6 mIgG compared to normal mouse IgG (1.5–1.8 fold). To directly investigate the *in vivo* phagocytic effect of antibodies, the mice were administered an i.p. injection of normal mouse IgG or 3F5H6 mIgG pre-incubated with FITC-labeled *S. aureus* MW2 cells. We then analyzed the phagocytic level by flow cytometry (Fig. 6H,I). Both the normal IgG and 3F5H6 mIgG enhanced the phagocytosis of macrophages (Fig. 6H) and dendritic cells (Fig. 6I) in the peritoneal cavity with higher effect by 3F5H6 mIgG than the normal IgG. These results suggested that 3F5H6 mIgG functions as an effective phagocytic mediator in mouse peritoneal cells and that phagocytic immune cells are involved in the antibacterial effect of CpG-induced antibodies.

### Therapeutic effects of the bacteria-reactive monoclonal antibody against *S. aureus* MW2 infection

**Figure 7:**
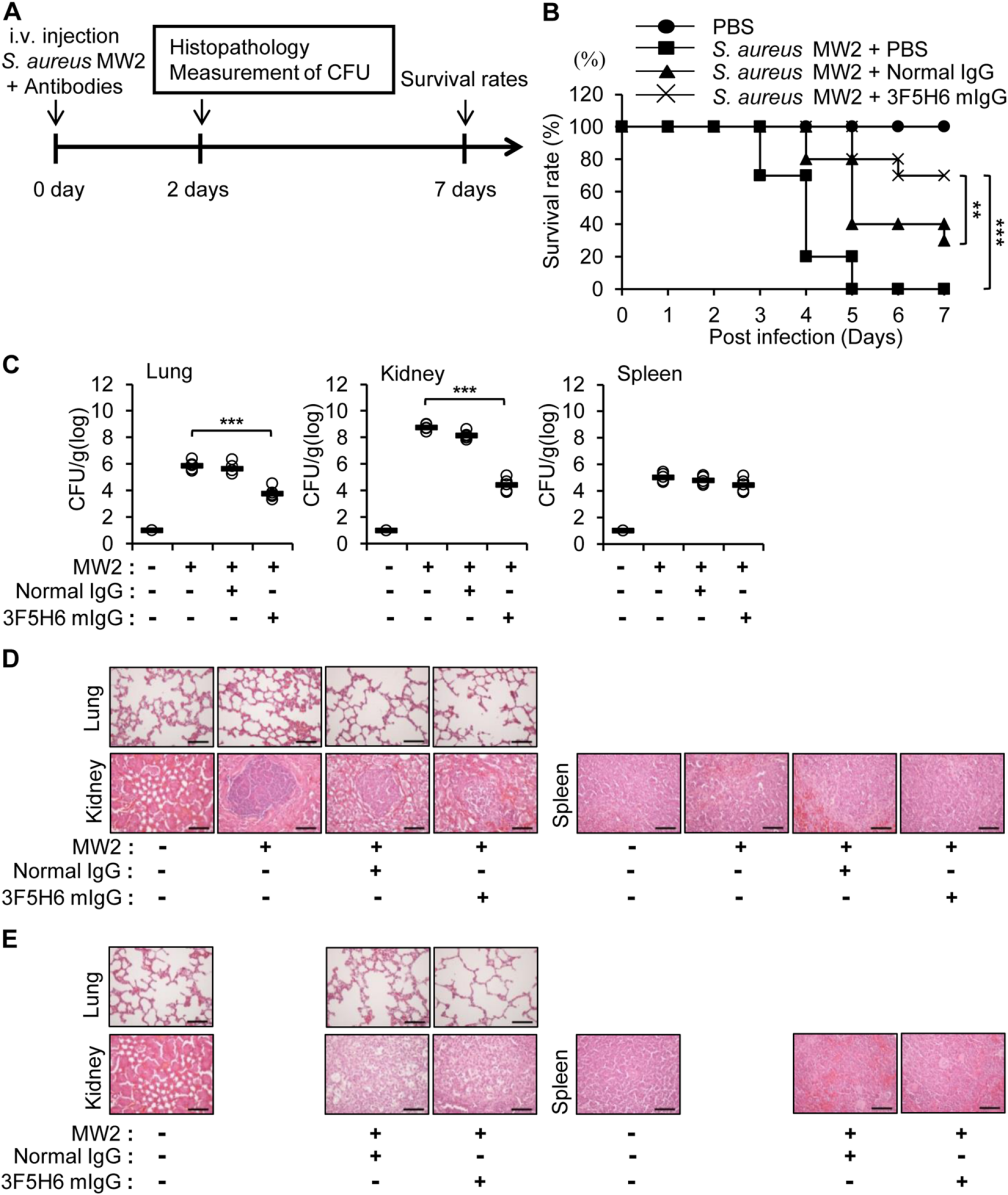
Effects of bacteria-reactive monoclonal antibody on survival of the *S. aureus* MW2-infected mice. **(A)** Schematic diagram of the experimental process. **(B)** Eight-week-old female BALB/c mice were i.v. injected with *S. aureus* MW2 (1.5 × 10^7^ CFU) and then subsequently i.v. injected with PBS, normal mouse IgG, or 3F5H6 mIgG (25 mg/Kg mouse), and survival rates were monitored for 7 days (n = 10/group). **(C)** Two days after *S. aureus* MW2 infection, *S. aureus* MW2 CFUs were determined in the indicated tissues (n = 5/group). **(D)** Histopathology of the indicated tissues 2 days after infection. **(E)** Histopathology of the indicated tissues 30 days after infection. Scale bar, 10 μm. MW2, *S. aureus* MW2. The results presented are representative of three experiments. **p < 0.005, ***p < 0.0005.

To confirm the antibacterial effects of 3F5H6 mIgG against *S. aureus* MW2 infection, BALB/c mice were infected with *S. aureus* MW2, administered an i.v. injection of PBS, normal IgG, or 3F5H6 mIgG, and then mortality, infection in tissues, and histopathology were observed according to the experimental schedule (Fig. 7A). All of the mice infected with *S. aureus* MW2 that did not receive antibody died 5 days after infection, but 30% of the normal IgG injected mice and 70% of 3F5H6 IgG injected mice survived until 7 days after infection (Fig. 7B). To investigate *S. aureus* MW2 infection in specific tissues, the lungs, kidneys, and spleen were prepared 2 days after infection and a CFU assay was performed. We observed decreased bacterial loads in the tissues, especially in the kidneys, from the mice administered the 3F5H6 mIgG (Fig. 7C). The histopathology of the tissues was also evaluated 2 days after infection. Bacterial burdens were only found in the kidney, and smaller bacterial burdens were detected in antibody-injected-mice compared to mice only infected with *S. aureus* MW2 (Fig. 7D). Thirty days after *S. aureus* MW2 infection, we examined the histopathology of the lungs, kidneys, and spleen, which revealed no bacterial burden and the infiltration of inflammatory immune cells in the kidneys of antibody-injected mice (Fig. 7E). When we injected antibodies 2 h after *S. aureus* MW2 infection, we obtained similar results even though the effect of antibody injection was milder (Supplementary Fig. S2). The final survival of the mice with delayed antibody injection was about 20% lower than the simultaneous injection group. Taken together, the results demonstrate that CpG-DNA-induced antibodies led to increased survival and enhanced bacterial clearance in *S. aureus* MW2-infected mice.

## Discussion

Bacterial DNA and synthetic oligonucleotides with CpG dinucleotide motifs (CpG-DNA) have diverse immunomodulatory effects. Further, the administration of CpG-DNA in *in vitro* and *in vivo* model systems has revealed protective effects against various bacterial species, including MRSA. In this study, we focused on the induction of bacteria-reactive antibodies following the administration of CpG-DNA and the underlying functional mechanisms of the antibodies against *S. aureus* infection. CpG-DNA triggered the production of bacteria-reactive antibodies in the peritoneal cavity, which significantly increased the survival of mice after *S. aureus* infection by enhancing phagocytosis.

CpG-DNA activates immune cells such as macrophages, dendritic cells, NK cells, and B lymphocytes^1,3,4,7^. CpG-DNA upregulates the expression of cytokines, chemokines, major histocompatibility (MHC) class II molecules, and costimulatory molecules in various immune cells^4–7^. Therefore, CpG-DNA are considered as potentially useful immune adjuvants^1,2,6^. Administering CpG-DNA to animals can enhance host immunity against intracellular and extracellular pathogens^15,16^. Here, we confirmed that the administration of CpG-DNA improved survival rates and enhanced bacterial clearance in tissues after *S. aureus* infection (Fig. 1). Several reports suggest that CpG-DNA pre-treatment within 72 h before infection induces cytokine expression, including IL-6, IL-12, IFN-γ, and TNF-α, which contribute to host defense^5,16,29^. However, the effects of cytokine expression might have been minimized in our study as we administered CpG-DNA 7 days before *S. aureus* infection. Therefore, we hypothesized that another mechanism was involved in the observed antibacterial effects of CpG-DNA.

CpG-DNA can trigger host immunity through phagocytosis^19,30^. An analysis of the cell populations in the mouse peritoneal cells revealed high percentages of myeloid cells in the peritoneal cavity of normal mice (Fig. 2A). Therefore, we speculated that many types of myeloid cells, such as macrophages, dendritic cells, and neutrophils, might be activated by CpG-DNA in the peritoneal cavity and promptly participate in the phagocytosis of the bacteria. Several studies have reported bacterial infections that were induced by the intranasal (i.n.), i.v., or i.p. injection of bacteria^9,26,29^. In particular, one study noted that almost 5 × 10^8^ CFU of *S. aureus* were needed to perform survival experiments in mice following i.p. infection^26^. Interestingly, this concentration of *S. aureus* is approximately 40–50 times higher than the concentration used for i.v. infection in this study, which supports the essential role of peritoneal cells in bacterial removal.

Two days after i.v. *S. aureus* infection, the number of lymphoid and myeloid cells was drastically decreased and the ratios of immune cells were altered in the peritoneal cavity, spleen, and bone marrow. The change in the cell populations was especially prominent in the peritoneal cavity. B2 and T cells, known as adaptive immune cells, were virtually nonexistent in mice infected with *S. aureus* without CpG-DNA pre-treatment. However, B2 and T cell populations were largely maintained in the mice pre-treated with CpG-DNA prior to *S. aureus* infection (Fig. 2A). Therefore, these data suggest that the preservation of immune cell populations is the first line of CpG-DNA-mediated antibacterial effects. Nonetheless, the mechanism underlying this observation remains unclear. Because the amount of total antibodies increased following CpG-DNA treatment (Fig. 3), we believe that CpG-DNA triggered the differentiation and expansion of some B cells, which then secreted antibodies, contributing to the antibacterial activity.

It has been reported that natural antibodies have antibacterial activity^31^. More specifically, it was suggested that some natural antibodies in normal serum, called polyreactive antibodies, have broad antibacterial activity and prevent the infection of several bacteria^32^. These bacteria-reactive antibodies have moderate binding affinity to a variety of antigens. Here, we confirmed that antibodies with broad reactivity against bacteria exist in the murine peritoneal cavity and serum in the absence of bacterial stimulation. In addition, we found that the amount of bacteria-reactive antibodies was markedly increased by the administration of CpG-DNA, especially in the peritoneal cavity (Fig. 3). Furthermore, the bacteria-reactive antibody levels were significantly increased when we isolated peritoneal B cells and stimulated them with CpG-DNA (Fig. 4). Therefore, we propose that peritoneal B cells might be the primary targets of CpG-DNA in our system, and that antibody production can be induced in a T cell-independent manner. Considering that serological memory can be maintained by polyclonal activation of human memory B cells^33^, it is one possibility that stimulation of TLR9 with CpG-DNA results in the proliferation of existing memory B cells. Previously, it was suggested that B1 cells predominantly reside in the peritoneal cavity and produce polyreactive antibodies in response to microbial antigens^34,35^. Therefore, we investigated whether CpG-DNA had a differential effect on the production of bacteria-reactive antibodies by B1 and B2 cells in the peritoneal cavity and found that both B cell types produced bacteria-reactive antibodies in response to CpG-DNA, although B1 cells generally produced higher antibody amounts than B2 cells. When we repeated the CpG-DNA injections in mice, the amount of total IgG and bacteria-reactive IgG was increased (data not shown). However, no enhanced effect was observed *in vitro* (Fig. 4). Therefore, it is likely that cellular or molecular factors other than B cells are involved in the enhanced effects of CpG-DNA observed *in vivo*.

The bacteria-reactive antibodies isolated from the peritoneal cavity of normal mice and CpG-DNA-treated mice enhanced phagocytosis in RAW 264.7 cells, with higher activity apparent with the antibodies from the CpG-DNA-treated mice (Fig. 5). When we examined the direct effects of normal IgG and the 3F5H6 mIgG bacteria-reactive monoclonal antibody isolated from CpG-DNA-stimulated peritoneal B cells, we found that the phagocytic activity of RAW264.7 cells and primary peritoneal cells were enhanced by the antibodies (Fig. 6). Further, the phagocytic activity of the cells was more effectively enhanced by 3F5H6 mIgG than normal IgG. As well, the injection of antibodies enhanced survival and bacterial clearance in several tissues in the *S. aureus*-infected mice, and 3F5H6 mIgG was much more effective than normal IgG (Fig. 7). Therefore, we suggest that CpG-DNA stimulates the production of bacteria-reactive antibodies, which, in turn, contribute to the defense against bacterial infection through mechanisms including enhanced phagocytosis, as previously suggested^32,36^. Because 3F5H6 mIgG exhibited superior effects compared to normal IgG, 3F5H6 mIgG might be a promising therapeutic for the treatment of bacterial infections.

The protective effects of CpG-DNA-induced antibodies following *S. aureus* infection was confirmed, but bacterial clearance was more evident in the kidney from CpG-DNA-injected mice than in those from antibody-injected mice (Figs 1C,D and 7C,D). A detailed investigation of antibody-injected mice that survived for 30 days after *S. aureus* infection revealed that the mice had no bacterial burden in the kidney and a greater number of immune cells in the kidney compared to PBS-injected mice, suggesting the efficient infiltration of inflammatory cells (Fig. 7E). Therefore, we believe that CpG-DNA could activate many factors, such as cytokines and other cellular mechanisms, in addition to the production of bacteria-reactive antibodies, and that together these factors might cause more efficacious immunomodulatory effects following bacterial infection. Although we focused on IgG in this study, natural polyreactive antibodies are mainly IgM^37^. IgM antibodies have lower affinity than IgG, but have advantages such as high avidity and broad specificity. Therefore, stimulation with CpG-DNA can activate more efficiently than the injection of IgG alone. Taken together, it is likely that CpG-DNA activates innate immunity and alerts the immune system, which then promotes a more efficient response. Some reports insist that a combined treatment with antibodies and antibiotics is more effective against MRSA bacteremia than one type of treatment^23,24^. In this context, combined therapies involving various combinations of antibodies, adjuvants, and antibiotics, could be considered.

In conclusion, we demonstrated that bacteria-reactive antibodies induced by CpG-DNA administration exhibit protective functions against *S. aureus* infection in murine models, and that the antibodies enhanced phagocytic efficiency. Although passive immunization has been the recent focus of numerous investigations, no antibodies have been approved for clinical use. Therefore, future studies should focus on the development of a humanized antibody to resist MRSA infection, which might also be useful in the treatment of infectious diseases.

## Methods

### Animals

Eight-week-old BABL/c mice (H-2^b^) were purchased from Nara Biotech, Inc. (Seoul, Korea) and BALB/c TLR9 knockout mice (TLR9^−/−^) were purchased from Oriental BioService, Inc. (Kyoto, Japan). The mice were maintained under specific-pathogen-free conditions (20–25 °C, 40–45% humidity, 12 h light/dark cycle; food and water access, *ad libitum*). All procedures for the animal experiments were performed according to the Guide for the Care and Use of Laboratory Animals of the National Veterinary Research and Quarantine Service of Korea with the approval of the Institutional Animal Care and Use Committee of Hallym University (Permit Number: Hallym 2014-66, 2015-54, 2016-22, 2016-36). Mice were anesthetized under isoflurane (3% to 5% for induction and 1% to 3% for maintenance, JW Pharmaceutical, Seoul, Korea) inhalation to minimize pain. After the experiments were completed, the mice were sacrificed by CO_2_ inhalation and all efforts were made to minimize suffering.

### CpG-DNA

CpG-DNA 1826 and non-CpG-DNA 2041 were purchased from GenoTech (Daejeon, Korea). The backbones of the sequences were modified with phosphorothioate. The following oligodeoxynucleotide sequences were used: CpG-DNA 1826, 5′-TCCATGACGTTCCTGACGTT-3′ and non-CpG-DNA 2041, 5′-CTGGTCTTTCTGGTTTTTTTCTGG-3′. The non-CpG-DNA 2041 was used as a negative control. Oligodeoxynucleotides were dissolved in distilled water.

### Bacteria culture and *in vivo* infection studies

*Staphylococcus aureus* (*S. aureus*, KCCM 12103), *Staphylococcus epidermidis* (S. *epidermidis*, KCCM 40416), and *Streptococcus pyogenes* (*S. pyogenes*, KCCM 11873) were purchased from the Korean Culture Center of Microorganisms (KCCM, Seoul, Korea). The *S. aureus* Δ*spa* mutant (M0107) was used. Strain M0107 is an IgG-binding protein A-deficient Δ*spa* mutant of RN4220^38^. All bacteria were grown at 37 °C in Lysogeny broth (LB). *S. aureus* strain MW2 (MRSA) was grown at 37 °C in Columbia broth supplemented with 2% NaCl^39^. All bacteria were grown overnight, re-cultured in fresh media at a 1/50 dilution until the mid-log phase (OD_600_ 0.5–0.6), and harvested. *S. aureus* MW2 was washed with PBS, centrifuged, and diluted to 5 × 10^7^ colony forming units (CFU)/mL in PBS. BABL/c mice were injected with PBS or 50 μg of CpG-DNA 1826 intraperitoneally (i.p.). After 7 days, the mice were injected i.v. with 0.2 ml of PBS or the bacterial suspension. Following infection, the mice were observed for morbidity or recovery for 7 days. We investigated the survival rate as well as the histopathology, bacterial loads (CFU), and immune cell populations in the control and infected mouse tissues, and measured antibody levels in the peritoneal fluid and serum from the control and infected mice.

### H&E staining

Paraffin embedding and tissue sectioning was performed using conventional methods^40^. After infection with *S. aureus* MW2, tissues including lung, kidney, and spleen were collected from the mice, prepared and mounted on slides, and dried at 40 °C overnight. The tissue slides were then incubated at 60 °C for 30 min to melt the paraffin. Next, the tissues were incubated in xylene, rehydrated through a series of 100–70% ethanol, and washed with distilled water. The tissues were then stained with Gill’s Hematoxylin V (Muto Pure Chemicals, Tokyo, Japan), washed with water, and stained with Eosin Y solution (Sigma-Aldrich, St. Louis, MO, USA). The stained tissues were dehydrated in a series of 70–100% ethanol, incubated in xylene, and mounted with Malinol medium (Muto Pure Chemicals). The stained tissues were observed using an Eclipse E200 microscope (Nikon, Japan).

### Analysis of colony forming units

Two days after infection, each tissue was harvested, weighed, and homogenized in PBS in a 2 mL Eppendorf tube (Eppendorf, Hamburg, Germany) containing stainless steel beads (Qiagen, Hilden, Germany). The homogenized solution was then transferred to 6-well plates containing Columbia broth-Bacto™ Agar, incubated overnight at 37 °C, and the number of colonies were counted.

### Preparation of serum, peritoneal cells, splenocytes, and bone marrow cells

Two days after infection with *S. aureus* MW2, the mice were anesthetized with isoflurane and sera were collected using the cardiac puncture method. Peritoneal cells, splenocytes, and bone marrow cells were harvested from the mice and cultured in RPMI 1640 medium containing 5% fetal bovine serum (FBS) as previously described^41–43^. After the cells were collected, the erythrocytes were removed using a red blood cell lysis buffer (140 mM NH_4_Cl, 20 mM Tris-HCl (pH 7.2)). The prepared cells were suspended in RPMI 1640 medium containing 5% FBS for stimulation with CpG-DNA *in vitro* and dispensed into 96-well tissue culture plates (BD Falcon, Falcon, Mexico).

### Flow cytometry

The cells prepared from the mice were blocked with anti-mouse CD16/32 (BD Biosciences, San Jose, CA, USA) for 10 min and stained with the following fluorescence-labeled antibodies: anti-mouse CD8, CD11c, CD3, CD4, CD11b, CD19 (BD Biosciences, USA), B220, CD23, F4/80, and Ly-6G antibodies (eBioscience, San Diego, CA, USA). The samples were then washed with PBS containing 1% FBS and analyzed using a FACSCanto^TM^ II system (Becton Dickinson, Franklin Lakes, NJ, USA).

### ELISA

To determine the production of bacteria-specific antibodies following CpG-DNA 1826 administration and/or *S. aureus* MW2 infection in mice, we used poly-L-lysine coated plates (Corning Inc, Corning city, NY, USA). The bacteria were grown overnight, washed twice with PBS by centrifugation at 10,000 rpm for 15 min, and re-suspended in ELISA coating buffer (15 mM Na_2_CO_3_, 35 mM NaHCO_3_, pH 9.6). Each well was coated with 100 μl of re-suspended bacteria (5 × 10^7^ CFU/well) and incubated overnight at 4 °C. After incubation, the bacteria were fixed with 0.5% glutaraldehyde in PBS for 15 min at room temperature. After washing twice with PBS, each well was incubated with RPMI 1640 medium containing 100 mM glycine and 0.1% BSA for 30 min at room temperature to block glutaraldehyde and washed twice with PBS. The bacteria-coated wells were then blocked with PBS containing 1% BSA for 1 h at room temperature. Serum, supernatants from the peritoneal cavity fluid or the peritoneal cell culture, or purified antibodies were serial diluted, added to each well, and incubated for 1 h at room temperature. The samples were washed three times with PBS-T (0.2% Tween-20 in PBS) and antibodies, including horseradish peroxidase (HRP)-labeled goat anti-mouse IgG (Jackson ImmunoResearch Inc, West Grove, PA, USA), IgG1, IgG2a, IgG2b, or IgG3 (Southern Biotech, Birmingham, AL, USA) were added to the wells and incubated for 1 h at room temperature. After washing with PBS-T four times, the TMB Microwell Peroxidase Substrate Kit (KPL, Gaithersburg, MD, USA) was used to detect peroxidase-labeled conjugates (blue-color expression), followed by the addition of TMB Stop solution (KPL) (yellow-color), and the absorbance was measured at 450 nm using a SpectraMax 250 microplate reader (Molecular Devices, Sunnyvale, CA, USA). To determine the antibody concentrations using ELISA, the plates were coated with goat anti-mouse IgG (Jackson ImmunoResearch Inc.) and incubated overnight at 4 °C. The wells were then blocked with PBS containing 1% BSA for 1 h at room temperature, and the amounts of total IgG and IgG isotypes were measured by ELISA as previously described^44^.

### *In vitro* stimulation of mouse peritoneal cells with CpG-DNA

Peritoneal cells were harvested from the mice using RPMI 1640 medium containing 5% FBS. After removing the erythrocytes, the cells were washed with RPMI 1640 medium containing 5% FBS and cultured in RPMI 1640 medium containing 5% FBS with 100 U/ml of penicillin and 100 μg/ml of streptomycin. Next, 5 μg/ml CpG-DNA 1826 was added to each cell culture plate. After 48 h, the cell culture supernatants were harvested and analyzed by ELISA to quantify the antibody levels.

### Sorting B cells from mouse peritoneal cells

The cells were stained with anti-mouse CD19 (BD Bioscience) to distinguish the B cells, and with anti-mouse CD23 (eBioscience) to sort the B1 from the B2 cells. Anti-mouse CD3 (BD Bioscience) was used to stain T cells. Peritoneal cells were stained with antibodies, washed, and suspended with sorting buffer (1 mM EDTA, 25 mM HEPES pH 7.0, 1% FBS diluted in PBS). B1 cells and B2 cells were sorted using a FACSAria^TM^ II system (Becton Dickinson).

### Purification of polyclonal antibodies from the mouse peritoneal cavity

The mice were administered PBS or CpG-DNA 1826 via an i.p injection. Seven days after administration, supernatants from the peritoneal cavity fluid were prepared by centrifugation to remove the peritoneal cells. Polyclonal antibodies were purified from the resulting supernatants using Protein A affinity chromatography (Repligen, Waltham, MA, USA) and analyzed by SDS-PAGE. The ability of these antibodies to bind *S. aureus* MW2 was measured by ELISA as described above.

### Production of hybridoma cells from B cells in the peritoneal cavity

To obtain hybridoma cells producing bacteria-reactive antibodies, BALB/c mice were i.p. injected with 50 μg of CpG-DNA 1826. Peritoneal cells were harvested from the mice after 7 days, fused with mouse SP2/0 myeloma cells, and bacteria-reactive antibody-producing hybridoma clones were screened using a standard hybridoma technique^45,46^. To obtain ascites, BALB/c mice were i.p. injected with the hybridoma clone after priming with pristine. After 9–11 days, ascites were harvested from the mouse peritoneal cavity. Monoclonal antibodies were purified from the ascites using Protein A affinity chromatography (Repligen) and analyzed by SDS-PAGE. The isotype and the bacteria-reactivity of the monoclonal antibody was measured by ELISA as described above.

### Fluorescent labeling of bacteria

*S. aureus* MW2 was grown to OD_600_ 0.5–0.6 (3 × 10^8^ CFU), harvested, washed, and fixed with 70% ethanol in PBS for 1 h. The fixed bacteria were labeled with 0.02 mM FITC (Sigma-Aldrich) in 0.1 M Na_2_CO_3_ buffer (pH 8.5) for 30 min at room temperature, washed with serum-free Hank’s Balanced Salt Solution (HBSS), and re-suspended in HBSS containing 2 mM CaCl_2_, 1 mM MgCl_2_, 10 mM HEPES, 150 mM NaCl, and 0.4% BSA.

### *In vitro* phagocytosis assays

The mouse RAW 264.7 macrophage cell line was purchased from American Type Culture Collection (ATCC, Manassas, VA, USA). The cells were cultured in Dulbecco’s Modified Eagle’s Medium (DMEM) with 10% FBS, 100 U/mL of penicillin, and 100 μg/mL of streptomycin. RAW 264.7 cells and mouse peritoneal cells were cultured overnight on poly-L-lysine-coated cover glass (Sigma) in 12-well plates (Nunc, Roskilde, Denmark). FITC-labeled *S. aureus* MW2 was incubated with PBS or antibodies for 1 h and then the bacteria were added to the 12-well plates. After 1 h incubation, the cells were fixed with 4% paraformaldehyde (Affymetrix, Santa Clara, CA, USA), washed with PBS, and stained with Hoechst No. 33258 (Sigma-Aldrich) at room temperature to identify cell nuclei. The mounted cells were analyzed using a LSM 710 laser scanning microscope (Carl Zeiss, Oberkochen, Germany). The phagocytic index was measured by counting the number of FITC-labeled *S. aureus* MW2 cells phagocytosed by RAW 264.7 cells and mouse peritoneal cells as previously described^47^.

### Uptake of *S. aureus* MW2 in the mouse peritoneal cavity

To determine the influence of antibodies on phagocytosis in the mouse peritoneal cavity, the mice were i.p. injected with FITC-labeled *S. aureus* MW2. After 1 h, peritoneal cells were harvested and stained with cell-specific markers including anti-F4/80, CD11b, and CD11c antibodies. The phagocytosis by macrophages and dendritic cells in the peritoneal cavity was measured by fluorescence-activated cell sorting (FACS) using a FACSCanto^TM^ II system (Becton Dickinson).

### Analysis of antibody effects on *S. aureus* MW2 infection *in vivo*

BABL/c mice were i.v. injected with 1.5 × 10^7^ CFU of *S. aureus* MW2, immediately followed by an i.v. injection of normal mouse IgG or monoclonal antibody (3F5H6 mIgG; 25 mg/kg mouse). Normal mouse IgG was purchased from Invitrogen (Carlsbad, CA, USA). After the injection of antibodies, survival rates were monitored for 7 days. Two days after *S. aureus* MW2 infection, the mice were sacrificed, the indicated tissues including lung, kidney, and spleen removed, and the number of *S. aureus* MW2 CFUs and histopathology were monitored.

### Statistical analysis

Results are presented as mean ± standard deviation. Differences between two samples were evaluated using a Student’s *t*-test, and a resulting value of *P* < 0.05 was considered statistically significant.

## Electronic supplementary material

Supplementary Information

## References

[1] Medzhitov R (2001). Toll-like receptors and innate immunity. Nat Rev Immunol.

[2] Krieg AM (2006). Therapeutic potential of Toll-like receptor 9 activation. Nat Rev Drug Discov.

[3] Messina JP, Gilkeson GS, Pisetsky DS (1991). Stimulation of *in vitro* murine lymphocyte proliferation by bacterial DNA. J Immunol.

[4] Ballas ZK, Rasmussen WL, Krieg AM (1996). Induction of NK activity in murine and human cells by CpG motifs in oligodeoxynucleotides and bacterial DNA. J Immunol.

[5] Klinman DM, Yi AK, Beaucage SL, Conover J, Krieg AM (1996). CpG motifs present in bacteria DNA rapidly induce lymphocytes to secrete interleukin 6, interleukin 12, and interferon gamma. Proc Natl Acad Sci USA.

[6] Carson DA, Raz E (1997). Oligonucleotide adjuvants for T helper 1 (Th1)-specific vaccination. J Exp Med.

[7] Krieg AM (1995). CpG motifs in bacterial DNA trigger direct B-cell activation. Nature.

[8] Bafica A (2005). TLR9 regulates Th1 responses and cooperates with TLR2 in mediating optimal resistance to Mycobacterium tuberculosis. J Exp Med.

[9] Carvalho NB (2011). Toll-like receptor 9 is required for full host resistance to Mycobacterium avium infection but plays no role in induction of Th1 responses. Infect Immun.

[10] Bhan U (2007). TLR9 is required for protective innate immunity in Gram-negative bacterial pneumonia: role of dendritic cells. J Immunol.

[11] Bhan U (2008). Toll-like receptor 9 regulates the lung macrophage phenotype and host immunity in murine pneumonia caused by Legionella pneumophila. Infect Immun.

[12] Noto MJ (2015). Toll-Like Receptor 9 Contributes to Defense against Acinetobacter baumannii Infection. Infect Immun.

[13] van der Meer, A.J. *et al*. Toll-like receptor 9 enhances bacterial clearance and limits lung consolidation in murine pneumonia caused by methicillin resistant Staphylococcus aureus. *Mol Med* **22** (2016). 10.2119/molmed.2015.00242 PMC5023514 27508882

[14] Ishii KJ (2005). CpG-activated Thy1.2+ dendritic cells protect against lethal Listeria monocytogenes infection. Eur J Immunol.

[15] Elkins KL, Rhinehart-Jones TR, Stibitz S, Conover JS, Klinman DM (1999). Bacterial DNA containing CpG motifs stimulates lymphocyte-dependent protection of mice against lethal infection with intracellular bacteria. J Immunol.

[16] Deng JC (2004). CpG oligodeoxynucleotides stimulate protective innate immunity against pulmonary Klebsiella infection. J Immunol.

[17] Lahiri A (2010). TLR 9 activation in dendritic cells enhances salmonella killing and antigen presentation via involvement of the reactive oxygen species. PLoS One.

[18] Mohamed W (2016). TLR9 mediates S. aureus killing inside osteoblasts via induction of oxidative stress. BMC Microbiol.

[19] Wu HM (2016). CpG-ODN promotes phagocytosis and autophagy through JNK/P38 signal pathway in Staphylococcus aureus-stimulated macrophage. Life Sci.

[20] McCaig LF, McDonald LC, Mandal S, Jernigan DB (2006). Staphylococcus aureus-associated skin and soft tissue infections in ambulatory care. Emerg Infect Dis.

[21] Adem PV (2005). Staphylococcus aureus sepsis and the Waterhouse-Friderichsen syndrome in children. N Engl J Med.

[22] Neu HC (1992). The crisis in antibiotic resistance. Science.

[23] Schaffer AC, Lee JC (2008). Vaccination and passive immunisation against Staphylococcus aureus. Int J Antimicrob Agents.

[24] Vernachio J (2003). Anti-clumping factor A immunoglobulin reduces the duration of methicillin-resistant Staphylococcus aureus bacteremia in an experimental model of infective endocarditis. Antimicrob Agents Chemother.

[25] Fattom AI, Horwith G, Fuller S, Propst M, Naso R (2004). Development of StaphVAX, a polysaccharide conjugate vaccine against S. aureus infection: from the lab bench to phase III clinical trials. Vaccine.

[26] Ohsawa H, Baba T, Enami J, Hiramatsu K (2015). Successful selection of an infection-protective anti-Staphylococcus aureus monoclonal antibody and its protective activity in murine infection models. Microbiol Immunol.

[27] Surewaard BG (2013). Staphylococcal alpha-phenol soluble modulins contribute to neutrophil lysis after phagocytosis. Cell Microbiol.

[28] Diep BA (2008). Contribution of Panton-Valentine leukocidin in community-associated methicillin-resistant Staphylococcus aureus pathogenesis. PLoS One.

[29] Judy BM (2012). Prophylactic application of CpG oligonucleotides augments the early host response and confers protection in acute melioidosis. PLoS One.

[30] Utaisincharoen P (2003). CpG ODN enhances uptake of bacteria by mouse macrophages. Clin Exp Immunol.

[31] Boes M, Prodeus AP, Schmidt T, Carroll MC, Chen J (1998). A critical role of natural immunoglobulin M in immediate defense against systemic bacterial infection. J Exp Med.

[32] Zhou ZH (2007). The broad antibacterial activity of the natural antibody repertoire is due to polyreactive antibodies. Cell Host Microbe.

[33] Bernasconi NL, Traggiai E, Lanzavecchia A (2002). Maintenance of serological memory by polyclonal activation of human memory B cells. Science.

[34] Hoffman W, Lakkis FG, Chalasani G (2016). B Cells, Antibodies, and More. Clin J Am Soc Nephrol.

[35] Cole LE (2009). Antigen-specific B-1a antibodies induced by Francisella tularensis LPS provide long-term protection against F. tularensis LVS challenge. Proc Natl Acad Sci USA.

[36] Panda S, Zhang J, Tan NS, Ho B, Ding JL (2013). Natural IgG antibodies provide innate protection against ficolin-opsonized bacteria. EMBO J.

[37] Boes M (2000). Role of natural and immune IgM antibodies in immune responses. Mol Immunol.

[38] Oku Y (2009). Pleiotropic roles of polyglycerolphosphate synthase of lipoteichoic acid in growth of Staphylococcus aureus cells. J Bacteriol.

[39] Brown S (2012). Methicillin resistance in Staphylococcus aureus requires glycosylated wall teichoic acids. Proc Natl Acad Sci USA.

[40] Kwon S (2012). Prevention and therapy of hepatocellular carcinoma by vaccination with TM4SF5 epitope-CpG-DNA-liposome complex without carriers. PLoS One.

[41] Fortier, A. H. & Falk, L. A. Chapter 14: Isolation of murine macrophages in *Current Protocols in Immunology* (ed. Coligan, J. E.) (John Wiley & Sons. Inc, Hoboken, NJ, 2001). 10.1002/0471142735.im1401s11 18432719

[42] Ray, A. & Dittel, B. N. Isolation of mouse peritoneal cavity cells. *J Vis Exp* (2010). 10.3791/1488 PMC3152216 20110936

[43] Stagg AJ, Burke F, Hill S, Knight SC (2001). Isolation of mouse spleen dendritic cells. Methods Mol Med.

[44] Wu G (2018). A Mucin1 C-terminal Subunit-directed Monoclonal Antibody Targets Overexpressed Mucin1 in Breast Cancer. Theranostics.

[45] Kim D (2011). Production of antibodies with peptide-CpG-DNA-liposome complex without carriers. BMC Immunol.

[46] Yokoyama, W. M., Christensen, M., Santos, G. D. & Miller, D. Chapter 2: Production of monoclonal antibodies in *Current Protocols in Immunology* (ed. Coligan, J. E.) (John Wiley & Sons. Inc, Hoboken, NJ, 2006). 10.1002/0471142735.im0205s74 18432969

[47] Sun R (2013). Hemocytic immune responses triggered by CpG ODNs in shrimp Litopenaeus vannamei. Fish Shellfish Immunol.

