# Supplementary material for: CpG-DNA exerts antibacterial effects by protecting immune cells and producing bacteria-reactive antibodies
Source: Sci Rep. 2018 Nov 2;8:16236. doi: 10.1038/s41598-018-34722-y (PMC6214913; doi:10.1038/s41598-018-34722-y)
Supplement: Supplementary file 1 — Supplementary Information [file 41598_2018_34722_MOESM1_ESM.pdf]

Supplementary information

CpG-DNA exerts antibacterial effects by protecting immune cells and producing bacteria-reactive antibodies

Authors: Te Ha Kim[1], Dongbum Kim[2], Avishekh Gautam[1], Heesu Lee[1], Min Hyung Kwak[1], Min Chul Park[1], Sangkyu Park[3], Guang Wu[2], Bok Luel Lee[4], Younghee Lee[3,*], Hyung-Joo Kwon[1,2,*]

Affiliations:

[1]Department of Microbiology, College of Medicine, Hallym University, Chuncheon, Republic of Korea

[2]Center for Medical Science Research, College of Medicine, Hallym University, Chuncheon, Republic of Korea

[3]Department of Biochemistry, College of Natural Sciences, Chungbuk National University, Cheongju, Republic of Korea

[4]Global Research Laboratory of Insect Symbiosis, College of Pharmacy, Pusan National University, Pusan, Republic of Korea

*Correspondence and requests for materials should be addressed to H.J.K (email: hjookwon@hallym.ac.kr) or Y.L. (email: yhl4177@cbnu.ac.kr)

Te Ha Kim and Dongbum Kim contributed equally to this work.

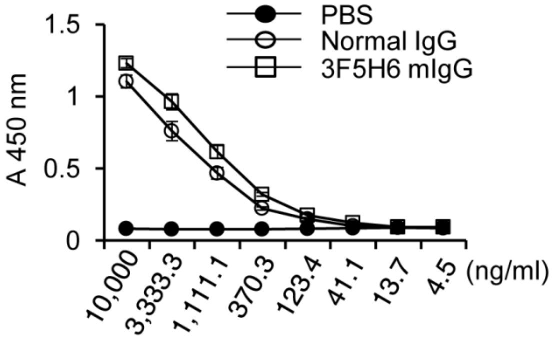

Supplementary Figure 1. Binding ability of the antibodies to *S. aureus* Δ*spa* mutant (M0107). Normal mouse IgG (normal IgG) and bacteria-reactive monoclonal antibody (3F5H6 mIgG) were captured using IgG-binding protein A-deficient *S. aureus* Δ*spa* (M0107) coated plates (n=3/group) and the binding ability was measured by ELISA.

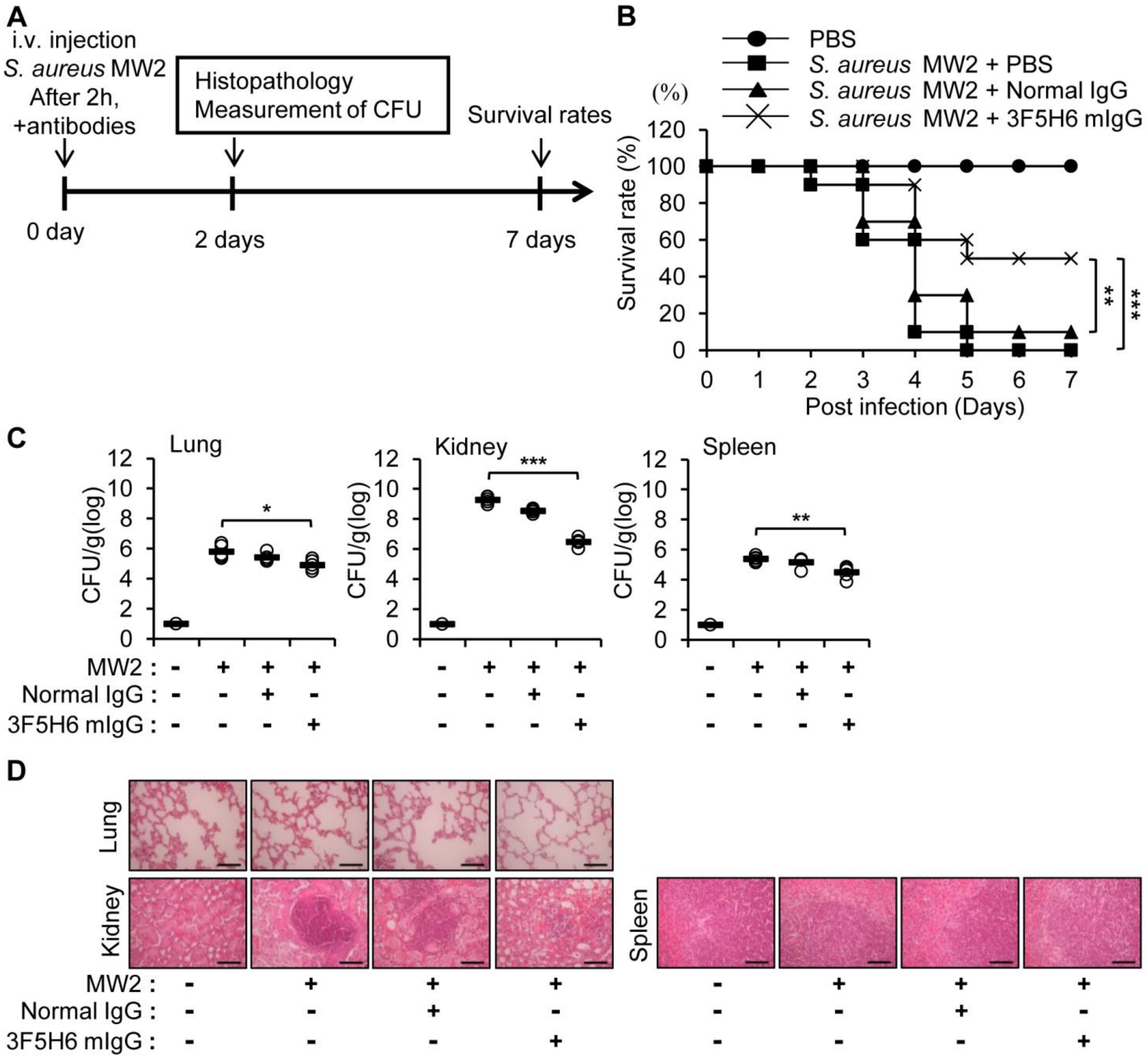

Supplementary Figure 2. Effects of bacteria-reactive monoclonal antibody on survival of the *S. aureus* MW2-infected mice. **(A)** Schematic diagram of the experimental process. **(B)** Eight-week-old female BALB/c mice were i.v. injected with *S. aureus* MW2 (1.5×10^7 CFU). After 2 h, the mice were i.v. injected with PBS, normal mouse IgG, or 3F5H6 mIgG (25 mg/Kg mouse), and survival rates were monitored for 7 days (n=10/group). **(C)** Two days after *S. aureus* MW2 infection, *S. aureus* MW2 CFUs were determined in the indicated tissues (n=5/group). **(D)** Histopathology of the indicated tissues 2 days after infection. Scale bar, 10 μm. MW2, *S. aureus* MW2. *$p < 0.05$, **$p < 0.005$, ***$p < 0.0005$.
